# NPC1L1 Drives Osteoporosis by Activating the C/EBPα/Cyp27a1/27‐Hydroxycholesterol Axis: A Novel Therapeutic Target for Bone Loss

**DOI:** 10.1096/fba.2025-00044

**Published:** 2025-05-08

**Authors:** Bohao Li, Wuling Zhou, Yueming Yu, Boyu Chen, Zhicheng Lv, Jiarui Zhang, Tieqi Zhang, Shiwei Sun, Lei Zhou, Minghai Wang

**Affiliations:** ^1^ Department of Orthopedics, Shanghai Fifth People's Hospital Fudan University Shanghai China; ^2^ Center of Community‐Based Health Research Fudan University Shanghai China; ^3^ Department of Orthopedic Surgery, Zhongshan Hospital Fudan University Shanghai China

**Keywords:** 27‐hydroxycholesterol, cholesterol metabolism, NPC1L1, osteogenic differentiation, osteoporosis

## Abstract

This study investigated how NPC1L1, a cholesterol transporter, regulates osteogenic differentiation through cholesterol metabolism independently of its transport function. We also explored the role of NPC1L1 in osteoporosis (OP), focusing on the downstream C/EBPα/Cyp27a1/27‐hydroxycholesterol (27‐OHC) axis. High‐throughput RNA sequencing and bioinformatics analysis identified NPC1L1 as a key regulator of osteogenesis. Osteogenic differentiation assays, Alizarin Red S and ALP staining, western blot analysis, and qRT‐PCR were performed using osteoblast cell lines (C3H10 and C2C12). In addition, an ovariectomy (OVX)‐induced mouse model of OP was established to validate the in vivo effects. ELISAs, chromatin immunoprecipitation (ChIP–qPCR), and rescue experiments were conducted to verify the functional interactions among NPC1L1, Cyp27a1, 27‐OHC production, and the transcription factor C/EBPα. NPC1L1 expression was downregulated during osteogenesis, and its knockdown significantly enhanced osteogenic differentiation, proliferation, and migration. At the molecular level, NPC1L1 promoted cholesterol metabolism independently of its transport function, resulting in elevated 27‐OHC levels through increased expression of Cyp27a1. Elevated 27‐OHC suppressed osteogenesis through the induction of oxidative stress and the downregulation of osteogenic biomarkers (ALP, OPN, OSX, and OCN). In OVX mice, NPC1L1 knockdown significantly reversed osteoporosis‐related bone loss, as evidenced by improved trabecular parameters (BV/TV%, Tb.Th, Tb.N). Furthermore, we identified C/EBPα as a transcriptional activator of Cyp27a1, which mediates the regulatory effects of NPC1L1 on 27‐OHC production. NPC1L1 inhibits osteogenesis and contributes to OP by promoting the Cyp27a1‐dependent synthesis of 27‐OHC through the transcription factor C/EBPα. Targeted modulation of the NPC1L1‐C/EBPα‐Cyp27a1‐27‐OHC axis could provide novel therapeutic strategies for OP.

## Graphical abstract

NPC1L1 Inhibits Osteogenesis via the C/EBPα/Cyp27a1/27‐OHC Axis in Osteoporosis

1. Mechanism:
  ○ NPC1L1 expression decreases during osteogenic differentiation.
  ○ NPC1L1 knockdown enhances osteoblast differentiation, proliferation, and migration.
  ○ NPC1L1 promotes cholesterol metabolism (independent of transport function), elevating Cyp27a1‐dependent 27‐hydroxycholesterol (27‐OHC) production via transcriptional activation by C/EBPα.
  ○ 27‐OHC induces oxidative stress, suppresses osteogenic biomarkers (ALP, OPN, OSX, OCN), and exacerbates bone loss.
2. In Vivo Validation:
  ○ NPC1L1 knockdown reverses osteoporosis in OVX mice, improving trabecular parameters (BV/TV%, Tb.Th, Tb.N).
3. Therapeutic Implication:
  ○ Targeting the NPC1L1/C/EBPα/Cyp27a1/27‐OHC axis (e.g., NPC1L1 inhibitors, Cyp27a1 antagonists) may restore bone homeostasis in osteoporosis.

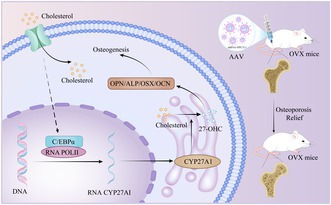

## 1 Introduction

Osteoporosis (OP) is a systemic metabolic bone disease characterized by decreased bone mass and degradation of the bone microarchitecture, which significantly increases the risk of fracture. The incidence of OP continues to rise annually as the global population ages [1, 2, 3]. The progression of OP is driven by multiple factors, including aging, genetic susceptibility, and imbalances in bone metabolism (characterized by reduced osteoblast‐mediated bone formation and increased osteoclast activity) [4, 5, 6]. The dynamic balance of bone metabolism relies upon the coordinated activities of osteoblast‐mediated bone formation and osteoclast‐mediated bone resorption. A key regulatory mechanism of bone homeostasis is the differentiation fate of bone marrow mesenchymal stem cells (BMSCs), which commit to either the osteogenic or the adipogenic lineage [7, 8, 9, 10]. Recent studies have further demonstrated that abnormalities in lipid metabolism, such as hypercholesterolemia, are strongly associated with OP pathogenesis [11, 12, 13]. Therefore, targeting lipid metabolic pathways, particularly cholesterol metabolism, may provide novel therapeutic strategies for the prevention and treatment of osteoporosis [14, 15].

Cholesterol, a lipid, has emerged in recent research as a significant contributor to the development and progression of OP, as hypercholesterolemia is an independent risk factor for decreased bone mass in postmenopausal women [16]. Cholesterol has been demonstrated to suppress the osteogenic differentiation capacity and proliferation of precursor MC3T3‐E1 osteoblasts. Moreover, it promotes osteoclast formation and enhances their bone‐resorbing function [17]. In support of this notion, the elevated expression of cholesterol 25‐hydroxylase (CH25H), a pivotal enzyme in cholesterol metabolism, in the femurs of ovariectomized mice with osteoporosis provides additional evidence for the regulatory role of cholesterol metabolism in bone metabolism [18]. Furthermore, several clinical studies have consistently demonstrated an inverse association between plasma total cholesterol (TC) levels, LDL‐C levels, and bone mineral density (BMD). Notably, serum HDL‐C levels exceeding 1.56 mmol/L significantly increase the risk of OP incidence [19]. Collectively, these observations indicate that cholesterol metabolism significantly influences OP progression. However, the exact molecular mechanisms underlying this association are not fully understood.

Niemann–Pick type C1‐Like 1 (NPC1L1) is a protein characterized by 13 transmembrane regions, a conserved N‐terminal “NPC1 domain,” and a sterol‐sensitive domain (SSD). As a critical transporter protein for cellular cholesterol absorption, NPC1L1 is also the target of the cholesterol‐lowering drug ezetimibe (Eze) [20, 21, 22]. Moreover, the expression level of NPC1L1 on the cell membrane is positively correlated with the human blood lipid content [23]. Liu et al. reported that osteopontin (OPN) downregulates NPC1L1 gene expression, leading to diminished cholesterol absorption and a subsequent reduction in gallstone incidence [24]. However, the specific role of NPC1L1 in OP remains unclear. In our previous investigation into the gene expression profile of circadian rhythm‐disrupted BMSCs, we observed that NPC1L1 expression was significantly correlated with inhibited osteogenic differentiation. Based on these findings, we designed additional cellular experiments and observed enhanced proliferation and osteogenic differentiation capabilities in NPC1L1‐knockdown osteoblasts.

In conclusion, given the existing evidence indicating a correlation between NPC1L1 expression and osteogenic differentiation, we hypothesize that NPC1L1 may play a regulatory role in osteogenesis. To test this hypothesis, the objectives of this study were to (1) characterize the dynamic expression pattern of NPC1L1 during osteogenic differentiation and its association with osteoporosis (RO1); (2) determine whether NPC1L1 regulates osteogenic activity through cholesterol metabolism independently of its transport function (RO2); and (3) elucidate the molecular mechanism of the NPC1L1/C/EBPα/Cyp27a1/27‐OHC axis (RO3).

## 2 Materials and Methods

### 2.1 Materials and Reagents

Alizarin Red S (AR‐S, A5533), an alkaline phosphatase detection kit (ALP Stain, SCR004), dimethyl sulfoxide (DMSO, D2650), dexamethasone (DXMS, D4902), L‐ascorbic acid (AA, A4403), β‐glycerophosphate (β‐GP, G9422), and dodecylpyridinium chloride (CPC, CDS000596) were purchased from Sigma–Aldrich (St. Louis, MO, USA). The primary antibodies used included anti‐NPC1L1 (NB400‐128, Novus, USA), anti‐β‐actin (AC026, ABclonal, China), anti‐GAPDH (AC001, ABclonal, China), anti‐C/EBPα (29388‐1‐AP, Proteintech, China), anti‐Cyp27a1 (14739‐1‐AP, Proteintech, China), anti‐ALP (A0514, ABclonal, China), anti‐OPN (22952‐1‐AP, Proteintech, China), anti‐OSX (A18699, ABclonal, China), anti‐OCN (16157‐1‐AP, Proteintech, China), anti‐Cyclin d1 (26939‐1‐AP, Proteintech, China), anti‐CDK4 (11026‐1‐AP, Proteintech, China), anti‐MMP2 (10373‐2‐AP, Proteintech, China), and anti‐MMP9 (10375‐2‐AP, Proteintech, China). The secondary antibodies used were goat anti‐rabbit IgG (7074, CST, USA) or anti‐mouse IgG (4410, CST, USA).

### 2.2 Cell Culture, Osteogenic Differentiation, and Cell Viability Assays via a Cell Counting Kit‐8 (CCK‐8)

C3H10, C2C12, and 293 T (used for lentiviral packaging) cell lines were obtained from the Cell Bank of the Chinese Academy of Sciences (Shanghai, China). The cells were cultured in high‐glucose Dulbecco's modified Eagle's medium (DMEM, HyClone) supplemented with 10% fetal bovine serum (FBS, Gibco).

For osteogenic differentiation, the basal culture medium was supplemented with dexamethasone (DXMS, 0.1 μM), L‐ascorbic acid (AA, 10 μM), and β‐glycerophosphate (β‐GP, 10 μM). Upon reaching approximately 80% confluence, the cultures were switched to an osteogenic induction medium. All the cell lines were incubated at 37°C in a humidified 5% CO_2_ atmosphere, and the media were changed every 2 days.

The cell proliferation rate was evaluated via the Cell Counting Kit‐8 (CCK‐8, Dojindo, Kumamoto, Japan) following the manufacturer's instructions.

### 2.3 Plasmids and Viral Infection

The PLKO.1‐EGFP‐puromycin, psPAX2, and pMD2.G were acquired from GeneChem (Shanghai, China) for lentiviral packaging and expression of short hairpin RNA (shRNA). Three sets of plasmids designed to suppress NPC1L1 expression are shown in Table 1. The full‐length coding sequence of NPC1L1 was cloned and inserted into the CMV‐MCS‐EGFP‐SV40‐Neomycin vector, which was subsequently transfected into target cells for NPC1L1 overexpression.

**TABLE 1:** Sequences of Thrap3‐shRNA.

Group	Sense strand	Antisense strand
NPC1L1‐shRNA1	5′‐ACAACAGAACAGTTTCATA‐3′	5′‐TATGAAACTGTTCTGTTGTGG‐3′
NPC1L1‐shRNA2	5′‐TGATCATAACCTTCTCTAT‐3′	5′‐ATAGAGAAGGTTATGATCAGG‐3′
NPC1L1‐shRNA3	5′‐TCCTTCAATACTTCCAGAA‐3′	5′‐TTCTGGAAGTATTGAAGGAGG‐3′
Control‐shRNA	5′‐TTCTCCGAACGTGTCACGT‐3′	

For lentivirus production, either the pLKO.1‐puro or pCDH‐CMV‐MCS‐EF1‐Puro vector was combined with the psPAX2 and pMD2.G and introduced into 293 T cells with 0.1% LipoFiter (LF‐1000, HanBio, Shanghai, China) in serum‐free DMEM. Twelve hours posttransfection, the medium was replaced with fresh DMEM containing 10% FBS. The lentiviral supernatants were collected at 48 and 72 h, filtered (0.45 μm cellulose acetate filter, Millipore, Billerica, USA), aliquoted, and stored at −80°C. Lentiviral infection of target cells was performed with 6 μg/mL polybrene. Stably transduced cells were selected with puromycin (3 μg/mL for 3 days, followed by 1 μg/mL for 2 weeks) and maintained in a medium containing 10% FBS.

### 2.4 Alkaline Phosphatase (ALP) and Alizarin Red S (AR‐S)

C3H10 and C2C12 cells were seeded in 24‐well plates and cultured with osteogenic induction medium. On days 0, 3, 6, 9, and 12, the media were removed, and the cells were washed twice with phosphate‐buffered saline (PBS) and fixed in 4% paraformaldehyde at 37°C for 10 min. The cells were then stained with ALP staining solution (SCR004, Sigma–Aldrich) or AR‐S staining solution (A5533, Sigma–Aldrich) at 37°C for 30 min, rinsed twice with PBS, air‐dried, and photographed. For quantification of AR‐S staining, the stained cells were incubated with 100 mM cetylpyridinium chloride (CPC) at 37°C for 50 min, and the absorbance at 562 nm was measured via a multiwell plate reader (Tecan Infinity 200Pro, Switzerland).

### 2.5 Quantitative Real‐Time PCR Protocol

Total RNA was extracted via an RNA‐Quick Purification Kit (SB‐R001, Share‐Bio, China). The RNA concentration and purity were measured with an Infinity 200‐Pro plate reader (Tecan, Switzerland). Complementary DNA (cDNA) synthesis was performed with PrimeScript RT Master Mix (RR036A, Takara, JPN).

The quantitative real‐time PCR (qRT–PCR) mixtures (10 μL total) contained 5 μL of 2× SYBR Premix Ex Taq (RR420A, Takara), 1 μL of diluted cDNA, and 0.2 μM primers. The reactions included initial denaturation at 95°C (10 min), 40 cycles of 95°C (15 s) and 55°C (34 s), and a final extension sequence. The experiments were repeated three times with triplicate samples. Gene expression was analyzed via the 2^−ΔΔCT method, with β‐actin serving as an internal control. The primer sequences are listed in Table 2 (from PrimerBank, https://pga.mgh.harvard.edu/primerbank/).

**TABLE 2:** Primer sequences.

Gene	Forward	Reverse
NPC1L1	5′‐TGTCCCCGCCTATACAATGG‐3′	5′‐CCTTGGTGATAGACAGGCTACTG‐3′
ALP	5′‐ACTGGGGCCTGAGATACCC‐3′	5′‐CCCGCTTTAACCAGTGCAAC‐3′
OPN	5′‐ATCTCACCATTCGGATGAGTCT‐3′	5′‐TGTAGGGACGATTGGAGTGAAA‐3′
OSX	5′‐GGAAAGGAGGCACAAAGAAGC‐3′	5′‐CCCCTTAGGCACTAGGAGC‐3′
OCN	5′‐CACTCCTCGCCCTATTGGC‐3′	5′‐CCCTCCTGCTTGGACACAAAG‐3′
β‐Actin	5′‐GGGACCTGACTGACTACCTC‐3′	5′‐TCATACTCCTGCTTGCTGAT‐3′

### 2.6 Protein Immunoblotting Procedure

The cells were washed twice with ice‐cold PBS and lysed in RIPA buffer (p0013b, Beyotime) containing 1% phenylmethylsulfonyl fluoride (PMSF, st506, Beyotime). The protein concentration was measured via a BCA protein assay kit (P0010, Beyotime). Proteins were separated by SDS–PAGE, transferred onto polyvinylidene fluoride (PVDF) membranes (Millipore), blocked, incubated with primary antibodies overnight at 4°C, and then incubated with secondary antibodies for 2 h at room temperature. Protein bands were visualized via enhanced chemiluminescence (ECL) detection (Share‐bio, China) and quantified via ImageJ software (version 1.8.0).

### 2.7 Construction of Animal Models and Group Administration

The animal experiments were approved by the Animal Welfare and Ethics Committee at Fudan University (Approval Date: 2023‐06‐10, Approval Number: 2023‐DWYY‐24JZS), and all the animal experiments were conducted in accordance with the ARRIVE guidelines. Specific pathogen‐free (SPF)‐grade female mice (20–22 g, 6–8 weeks old) were obtained from SPF Biotechnology Co. Ltd. (Beijing, China). The animals were randomly assigned to four groups: the Sham, OVX, OVX+Eze, and OVX+NPC1L1‐sh groups. Ovariectomy (OVX) surgery was performed, and adenovirus or drug treatment was initiated three weeks post‐surgery as specified.

### 2.8 Micro‐Computed Tomography (Micro‐CT) Analysis of Mouse Femurs

Mouse femurs were fixed overnight in 4% paraformaldehyde, scanned via micro‐CT (Bruker), and analyzed with NRvecon 1.6 and CTAn v1.13.8.1 software. The parameters included the bone volume/tissue volume (BV/TV%), trabecular thickness (Tb.Th), trabecular separation (Tb.Sp), and trabecular number (Tb.N).

### 2.9 Chromatin Immunoprecipitation (ChIP)

ChIP was performed via Pierce Chromatin Prep and ChIP kits (26158, Thermo Fisher Scientific) following the manufacturer's instructions. Immunoprecipitated DNA was purified for PCR analysis.

### 2.10 Statistical Analysis

Statistical analysis was performed via SPSS 25.0 (IBM Corp., Armonk, NY, USA). Continuous data are expressed as the mean ± standard deviation (SD) after confirming normality via the Shapiro–Wilk test. Independent groups were compared via Student's *t* test (two groups) or one‐way analysis of variance (ANOVA) (more than two groups), with homogeneity of variance verified via Levene's test. For ANOVA with significant main effects, Tukey's post hoc test was applied to control for the familywise error rate. A two‐tailed *p* value < 0.05 was considered statistically significant.

## 3 Results

### 3.1 NPC1L1 Is Downregulated During Osteogenesis and Significantly Correlated With Osteoporosis (RO1)

**FIGURE 1:**
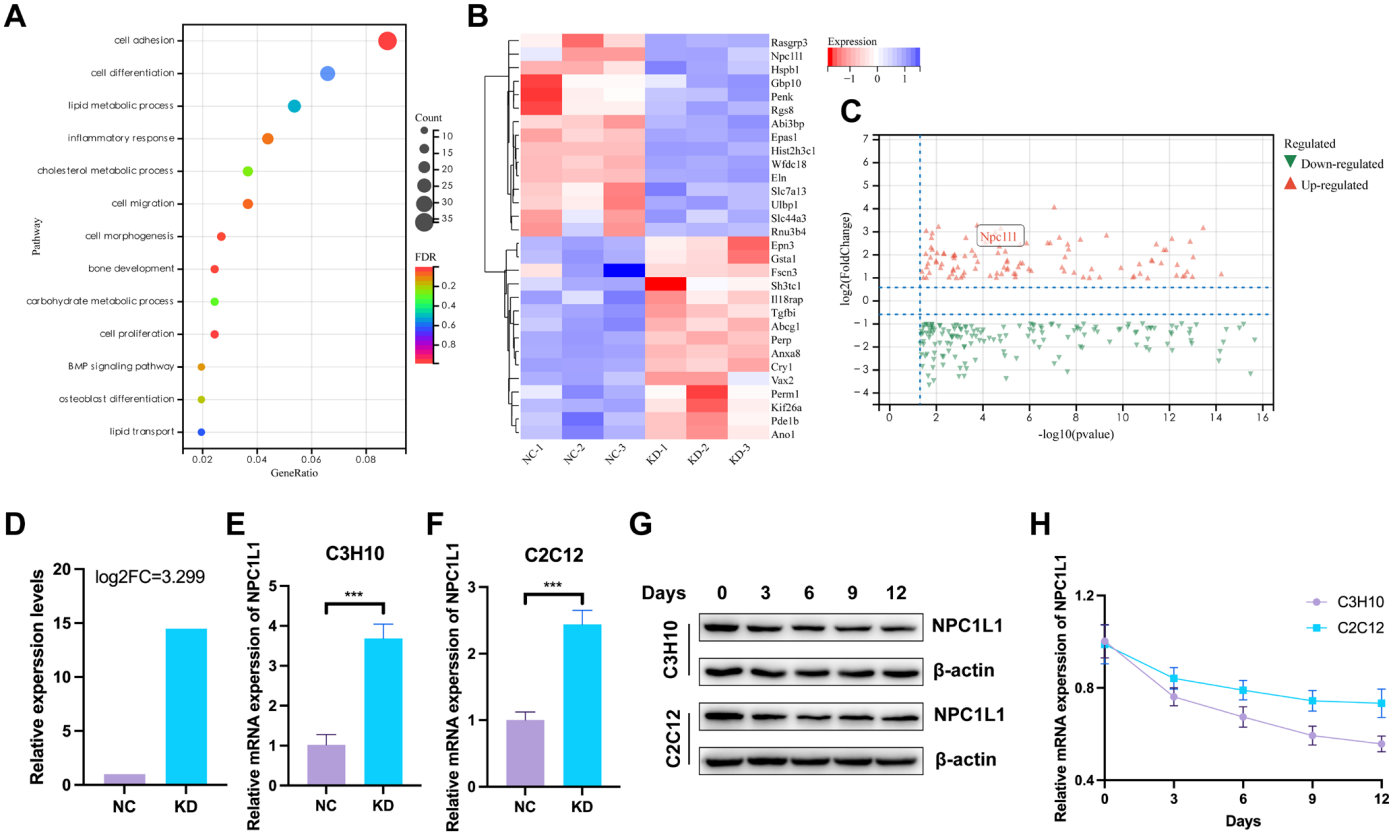
NPC1L1 expression is negatively associated with osteogenic differentiation. Gene ontology analysis (A) and heatmap (B) of dysregulated genes identified with RNA‐seq in control (NC) and Cry1 knockdown (KD) BMSCs. *n* = 3 biological replicates. Volcano plot (C) and relative expression level (D) of NPC1L1 expression in the RNA‐seq results. RT–qPCR analysis of NPC1L1 mRNA expression in control (E) and Cry1‐knockdown cell lines (F). (G) Representative western blot analysis of NPC1L1 protein levels in C3H10 and C2C12 cells treated with osteogenic medium for 0, 3, 6, 9, and 12 days. RT–qPCR analysis of NPC1L1 mRNA expression in C3H10 and C2C12 cells treated with osteogenic medium for 0, 3, 6, 9, and 12 days (H). All the data in the bar graphs are presented as the means ± SDs. *N* = 3 independent experiments. *p < 0.05; **p < 0.01; ***p < 0.001.

In our previous study, we demonstrated that the circadian clock regulates osteogenic differentiation and contributes to osteoporosis progression. We showed that Cryptochrome 1 (Cry1), a core circadian gene, promotes osteogenic differentiation. To further clarify the downstream mechanisms involved, we performed high‐throughput sequencing of Cry1‐knockdown BMSCs. Gene Ontology (GO) analysis of the differentially expressed genes revealed significant enrichment in lipid and cholesterol metabolic processes, which was consistent with previous research (Figure 1A). The heatmap (Figure 1B) and volcano plot (Figure 1C) highlighted the cholesterol transporter protein Niemann–Pick C1‐like 1 (NPC1L1) as one of the most markedly upregulated genes, with expression increased by more than tenfold compared with that of the controls (Figure 1D). Similarly, the gene expression profiles of femurs from postmenopausal osteoporosis patients revealed significantly increased NPC1L1 expression (GSE230665, Figure S1). RT–qPCR confirmed that NPC1L1 mRNA levels were significantly elevated in Cry1‐silenced C3H10 and C2C12 osteoblasts (Figure 1E,F). Western blot (Figure 1G) and RT–qPCR (Figure 1H) analyses revealed a negative correlation between NPC1L1 expression and osteogenic induction. Moreover, NPC1L1 expression tended to decrease between days 3 and 12 following osteogenic induction (Figure 1H), suggesting that it plays an early role in the osteogenic differentiation process. Taken together, these findings indicate that NPC1L1 is an important inhibitory factor in osteogenic differentiation during osteoporosis.

### 3.2 Loss of NPC1L1 Promotes Osteogenesis, Proliferation, and Migration (RO1)

**FIGURE 2:**
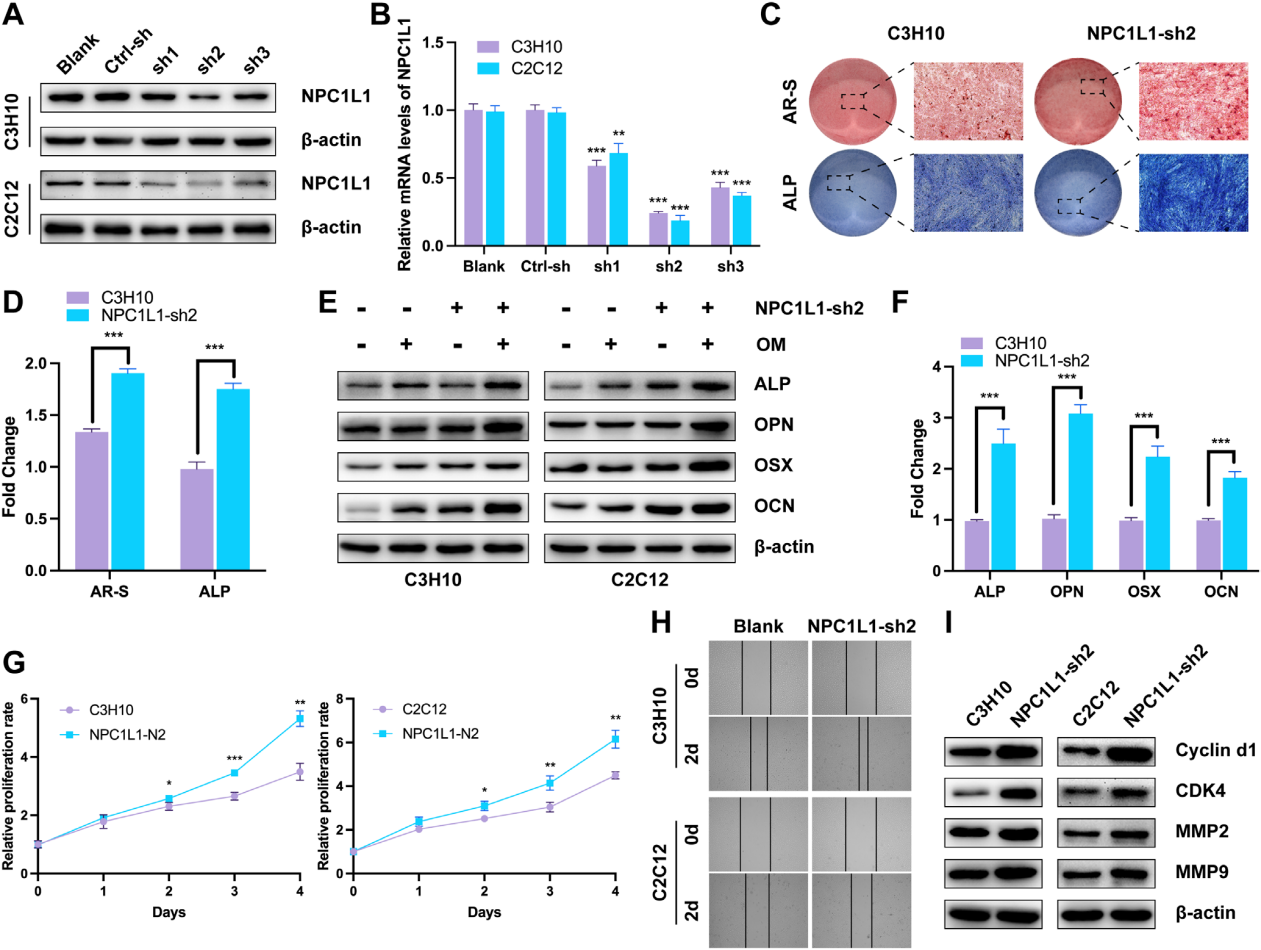
NPC1L1 knockdown enhances the osteogenesis, proliferation, and migration of osteoblast cells. Representative western blot (A) and RT–qPCR (B) analyses of NPC1L1‐knockdown cell lines. AR‐S (upper panel) and ALP (lower panel) staining of blank and NPC1L1‐sh2 C3H10 cells after induction with osteogenic medium for 3 days (C). Quantitative analysis of the AR‐S and ALP staining results is shown in Figure 2C (D). Representative western blot (E) and RT–qPCR (F) analyses of osteogenic biomarker genes (ALP, OPN, OSX, and OCN) in control and NPC1L1‐sh2 osteoblasts after induction with osteogenic medium for 3 days. CCK‐8 (G) and wound healing (H) assays of blank and NPC1L1‐sh2 osteoblast cells. Representative western blot analysis of biomarkers related to proliferation (Cyclin d1 and CDK4) and migration (MMP2 and MMP9) (I). All the data in the bar graphs are presented as the means ± SDs. *N* = 3 independent experiments. *p < 0.05; **p < 0.01; ***p < 0.001.

To determine the role of NPC1L1 in osteogenesis and osteoporosis progression, we used a lentiviral system to introduce shRNA‐expressing plasmids into the osteoblast C3H10 and MC3T3‐E1 cell lines. To ensure the efficiency of NPC1L1 knockdown, three shRNA sequences were tested. Western blotting and RT–qPCR revealed the greatest reduction in NPC1L1 expression at both the protein and mRNA levels in the NPC1L1‐sh2 group (Figure 2A,B). Therefore, shRNA‐2 (NPC1L1‐sh2, hereafter referred to as NPC1L1‐sh) was selected for subsequent experiments.

Next, we assessed the role of NPC1L1 in osteogenic differentiation. Compared with those in the control group, osteoblasts in the NPC1L1‐sh group responded to osteogenic induction, as indicated by increased Alizarin Red S (AR‐S) and ALP staining after 3 days (Figures 2C,D and S2). Consistent with this observation, the levels of the osteogenic biomarkers ALP, OPN, OSX, and OCN (protein and mRNA) were elevated, confirming enhanced osteogenic differentiation in NPC1L1‐sh cells (Figure 2E,F).

Moreover, NPC1L1‐sh cells exhibited faster proliferation and migration rates during culture. CCK‐8 and wound healing assays revealed significantly higher rates of cell growth and migration than control cells (Figure 2G,H). Accordingly, biomarkers for proliferation (Cyclin d1 and CDK4) and migration (MMP2 and MMP9) were increased in NPC1L1‐knockdown osteoblasts (Figure 2I). These findings suggest that NPC1L1 knockdown enhances the osteogenic differentiation, proliferation, and migration of osteoblasts.

**FIGURE 3:**
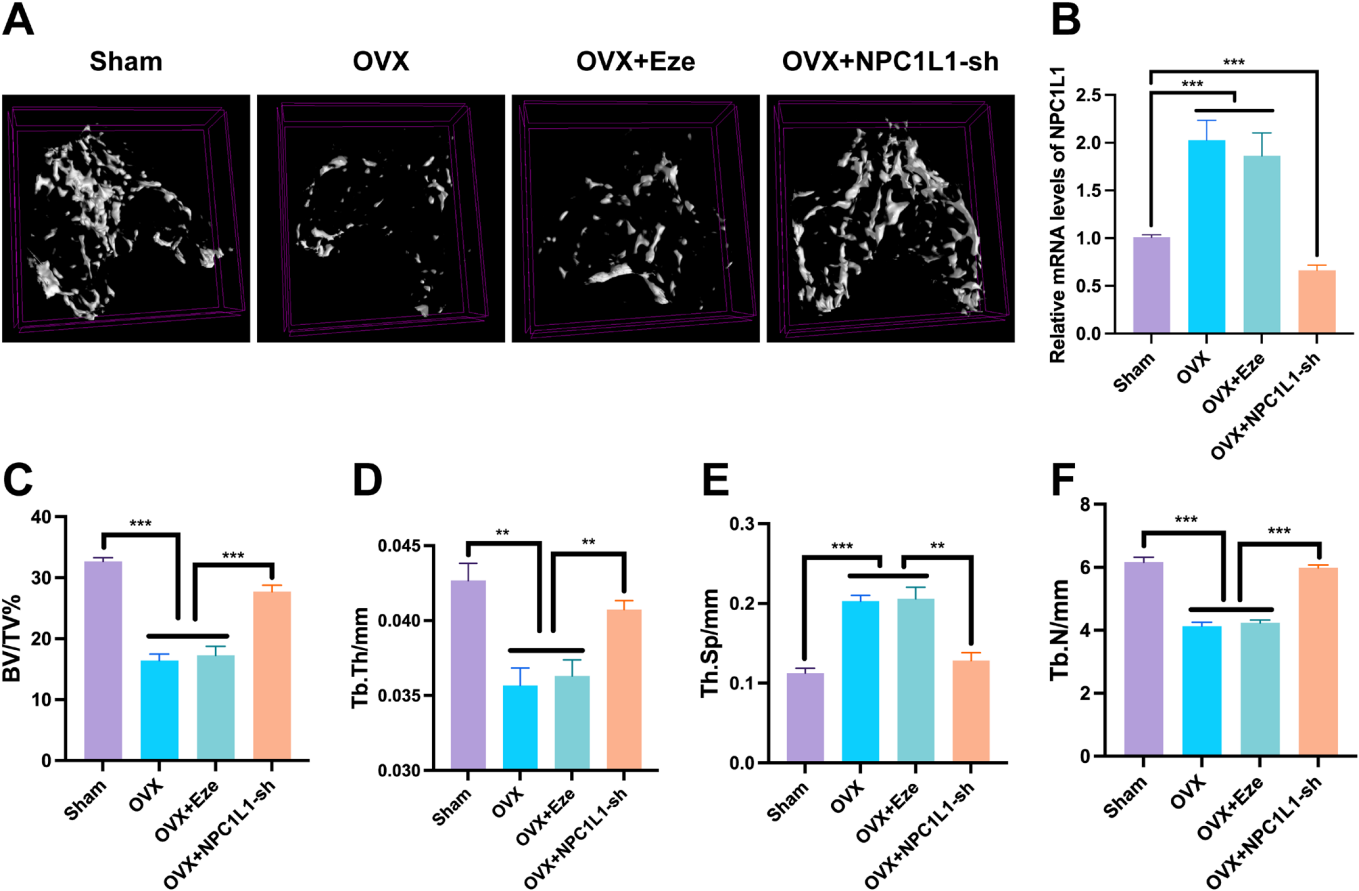
NPC1L1 knockdown rescues bone loss in OVX mice by improving trabecular microarchitecture. Micro‐CT images of the femurs of the sham‐operated, OVX, OVX+Eze, and OVX+NPC1L1‐shAAV mice (A). Relative mRNA expression in the BMSCs from the sham‐operated, OVX, OVX+Eze, and OVX+NPC1L1‐sh AAV mice (B). The bone volume per tissue volume (BV/TV%), trabecular thickness (Tb.Th), trabecular separation (Tb.Sp), and trabecular number (Tb.N) data are shown in Figure 3A (C–F). All the data in the bar graphs are presented as the means ± SDs. *n* = 5 mice per group. *p < 0.05; **p < 0.01; ***p < 0.001.

We subsequently used an adeno‐associated virus (AAV) to knock down NPC1L1 in the BMSCs of ovariectomized (OVX) mice. Three months later, three‐dimensional reconstruction images and cross‐sectional views of femurs obtained through micro‐CT revealed that NPC1L1 knockdown effectively reversed the osteoporotic trend in OVX mice, leading to increased bone formation compared with that in the sham‐operated controls (Figure 3A,B). In contrast, this reversal was not observed in OVX mice treated with Eze. Consistent with these findings, measurements of the bone volume‐to‐tissue volume ratio (BV/TV), trabecular thickness (Tb.Th), trabecular separation (Tb.Sp), and trabecular number (Tb.N) further confirmed that NPC1L1 knockdown progressively enhanced bone formation and delayed osteoporosis progression in OVX mice (Figure 3C–F).

### 3.3 NPC1L1 Inhibits Osteogenic Activity by Regulating Cholesterol Metabolism Independently of Its Transport Function (RO2)

**FIGURE 4:**
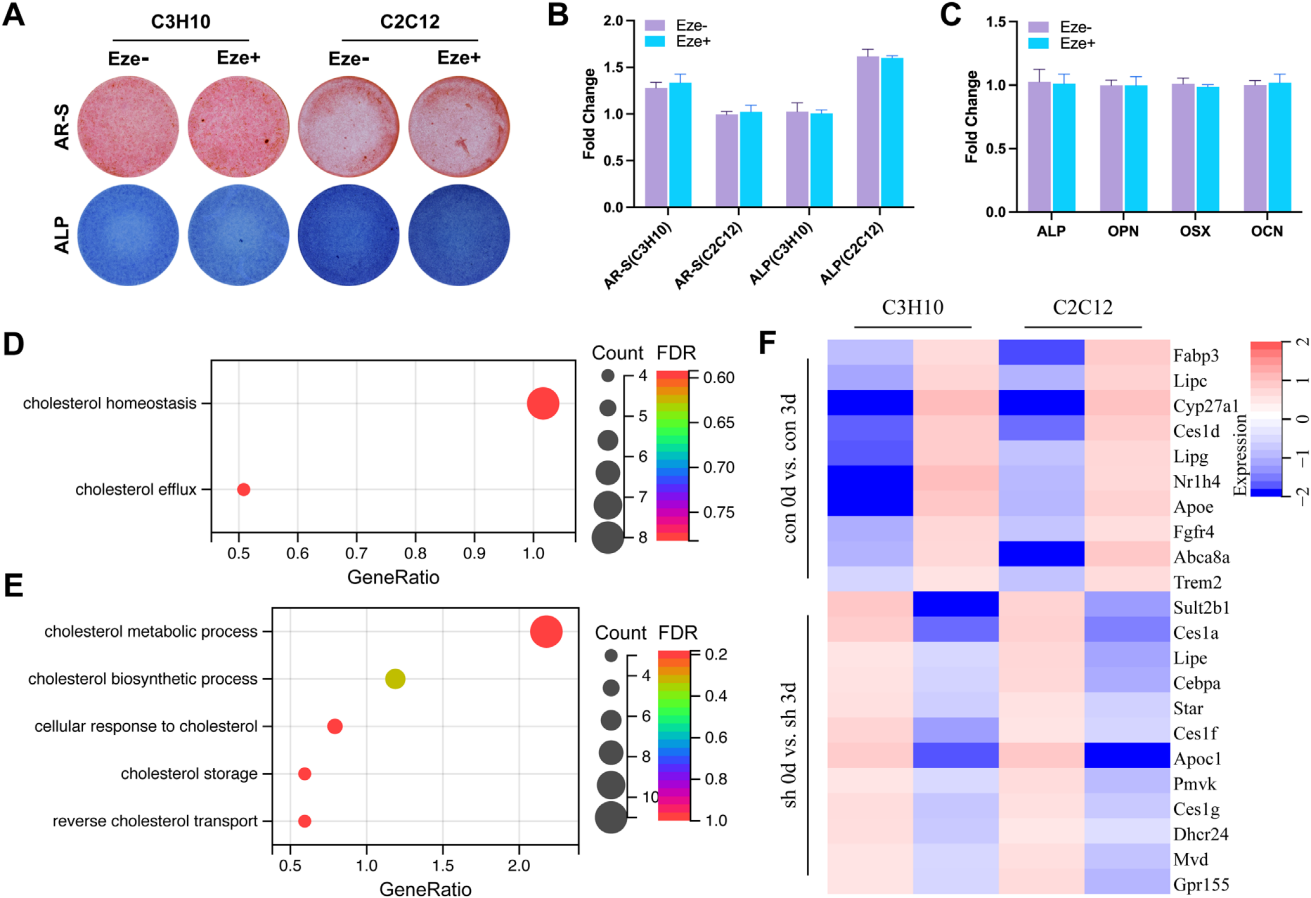
NPC1L1 regulates osteogenic differentiation through cholesterol metabolism. AR‐S (upper panel) and ALP (lower panel) staining of C3H10 and C2C12 cells treated with or without the NPC1L1 inhibitor Eze (A). Quantitative analysis of the AR‐S and ALP staining results is shown in Figure 3A (B). RT–qPCR analysis of osteogenic biomarker genes (ALP, OPN, OSX, and OCN) in C3H10 cells treated with or without the NPC1L1 inhibitor Eze (C). Gene ontology analysis of upregulated genes identified via RNA‐seq in blank C3H10 cells after induction with osteogenic medium for 3 days (D). Gene ontology analysis of downregulated genes identified via RNA‐seq in NPC1L1‐sh C3H10 cells after induction with osteogenic medium for 3 days (E). RT–qPCR analysis of the mRNA expression of cholesterol metabolism‐related genes in osteoblast cells after induction with osteogenic medium for 3 days (F). *N* = 3 independent experiments.

To determine whether NPC1L1 regulates osteogenic activity through cholesterol metabolism independently of its transport function (RO2), we used Eze, a specific inhibitor of NPC1L1‐mediated cholesterol transport, to block its transport function. We treated C3H10 and C2C12 cells with Eze. AR‐S and ALP staining (Figure 4A,B), as well as the mRNA expression of the osteogenic biomarkers ALP, OPN, OSX, and OCN (Figure 4C), revealed no significant differences after induction, indicating that Eze alone does not affect osteogenic activity.

To further identify the molecular mechanism underlying NPC1L1‐mediated osteogenesis, RNA sequencing (RNA‐seq) was performed on control and NPC1L1‐sh C3H10 cells at days 0 and 3 postinduction. GO analysis revealed that cholesterol metabolism process genes were significantly enriched among genes upregulated in control cells after induction (con 3d vs. con 0d, Figure 4D) but downregulated in NPC1L1‐sh cells after induction (sh 3d vs. sh 0d, Figure 4E). RT–qPCR validation (Figure 4F) supported these findings, confirming the regulatory role of NPC1L1 in cholesterol metabolism, but not cholesterol transport, during osteogenesis.

### 3.4 27‐OHC Production Mediates the Inhibitory Effect of NPC1L1 on Osteogenesis (RO3)

**FIGURE 5:**
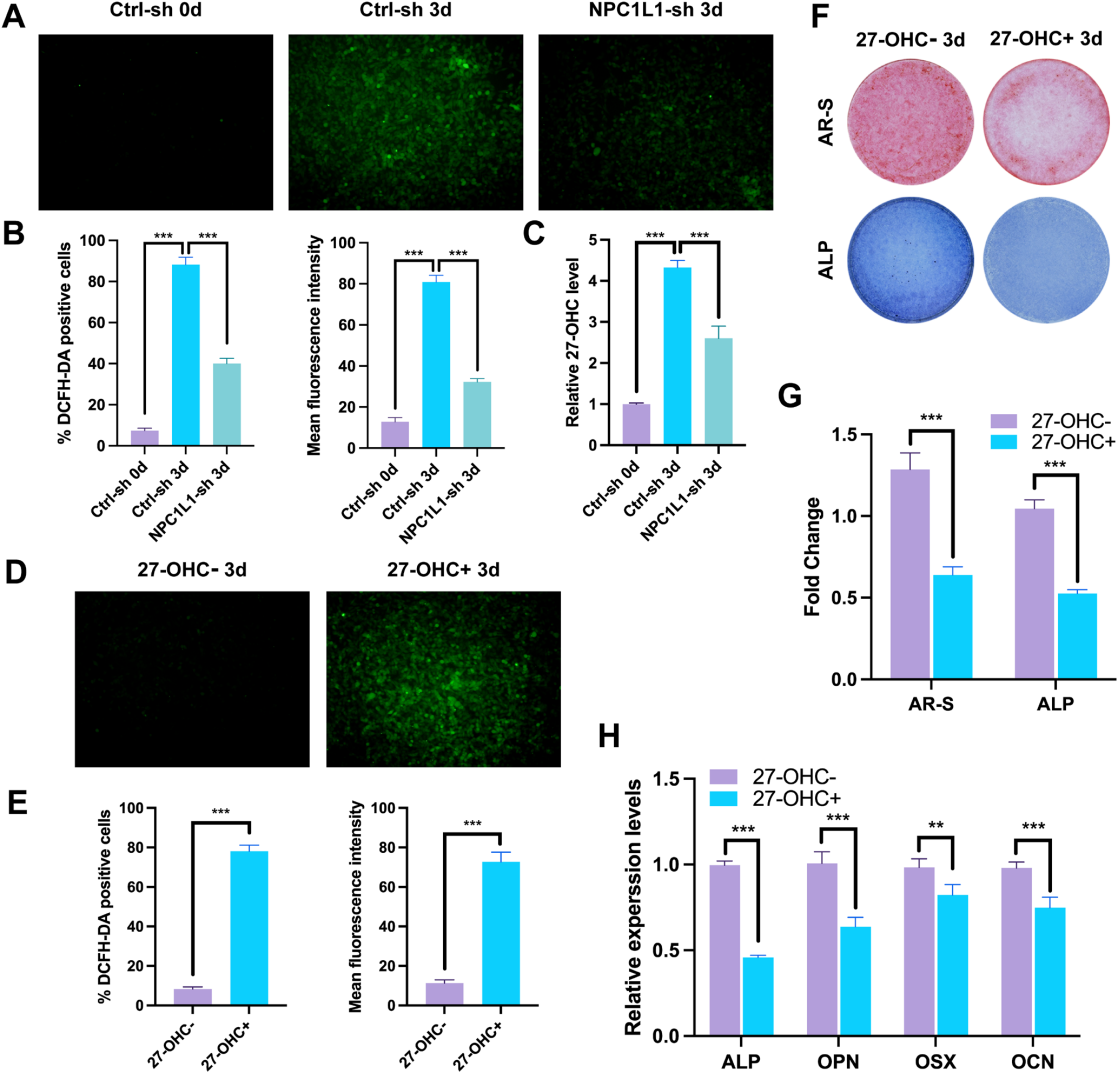
NPC1L1 induces 27‐OHC accumulation during osteogenic differentiation. Ctrl‐sh and NPC1L1‐sh C3H10 cells after induction with osteogenic medium for 3 days, as well as Ctrl‐sh C3H10 cells without osteogenic medium, were treated with the ROS probe DCFH‐DA (A). The results of the quantitative analysis of the percentage of DCFH‐DA‐positive cells and the mean fluorescence density are shown in Figure 4A (B). 27‐OHC levels were determined by ELISA (C). NPC1L1‐sh cells, with or without 27‐OHC treatment after induction with osteogenic medium for 3 days, were treated with the ROS probe DCFH‐DA (D). The results of the quantitative analysis of the percentage of DCFH‐DA‐positive cells and the mean fluorescence density are shown in Figure 4D (E). AR‐S and ALP staining of C3H10 cells with or without 27‐OHC treatment after induction with osteogenic medium for 3 days (F, G). RT–qPCR analysis of osteogenic biomarker genes (ALP, OPN, OSX, and OCN) in C3H10 cells treated with or without 27‐OHC (H). All the data in the bar graphs are presented as the means ± SDs. *N*=3 independent experiments. *p < 0.05; **p < 0.01; ***p < 0.001.

To clarify the role of cholesterol metabolism in NPC1L1‐mediated osteogenic inhibition, we measured reactive oxygen species (ROS) levels via DCFH‐DA fluorescence. The control osteoblasts presented significantly greater oxidation after 3 days of induction, whereas the NPC1L1‐sh osteoblasts presented markedly lower ROS levels (Figure 5A–C). ELISAs further revealed that the cholesterol oxidation product 27‐hydroxycholesterol (27‐OHC) was closely correlated with the ROS level (Figure 5D,E). Exogenous addition of 27‐OHC significantly increased ROS production and inhibited osteogenic differentiation, as evidenced by reduced AR‐S and ALP staining (Figure 5F,G) and decreased expression of the osteogenic markers ALP, OPN, OSX, and OCN (Figure 5H).

### 3.5 NPC1L1 Promotes Cyp27a1 Expression Through C/EBPα to Increase 27‐OHC Production (RO3)

**FIGURE 6:**
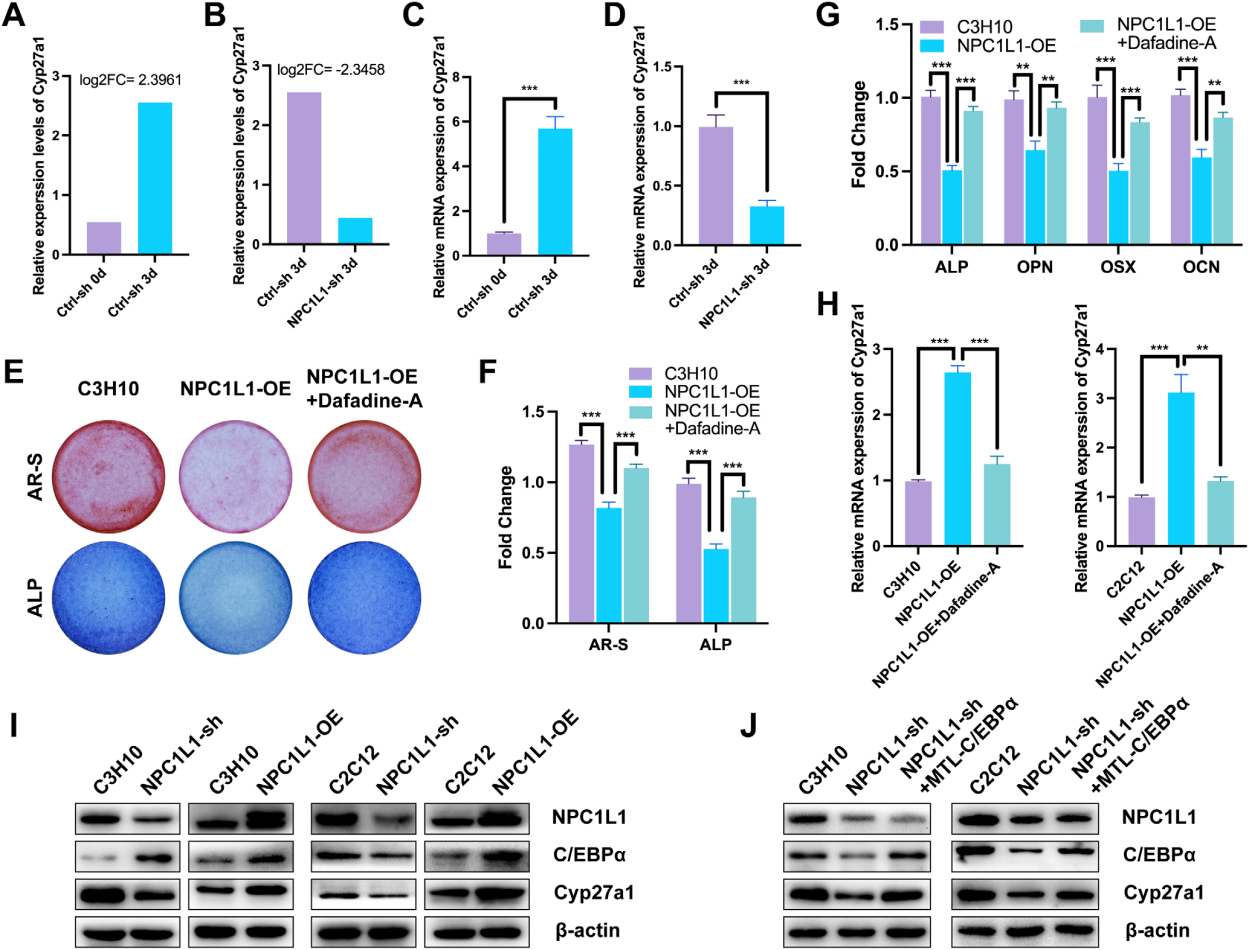
The С/ЕВР/Cyp27a1/27‐OHC axis mediates NPC1L1‐induced osteogenic inhibition. Relative expression levels determined by RNA‐seq of Ctrl‐sh or NPC1L1‐sh cells after induction with osteogenic medium for 3 days (A, B). RT–qPCR analysis of Cyp27a1 mRNA expression in Ctrl‐sh or NPC1L1‐sh cells after induction with osteogenic medium for 3 days (C, D). AR‐S (upper panel) and ALP (lower panel) staining of control, NPC1L1‐overexpressing (NPC1L1‐OE), and dafadine‐A‐treated NPC1L1‐OE C3H10 cells after induction with osteogenic medium for 3 days (E). The results of the quantitative analysis of the Alizarin Red S (AR‐S) and alkaline phosphatase (ALP) staining results are presented in Figure 6E (F). RT–qPCR analysis of osteogenic biomarker genes (ALP, OPN, OSX, and OCN) in control, NPC1L1‐overexpressing (NPC1L1‐OE), and dafadine‐A‐treated NPC1L1‐OE C3H10 cells after induction with osteogenic medium for 3 days (G). Relative Cyp27a1 mRNA expression in control, NPC1L1‐overexpressing (NPC1L1‐OE), and dafadine‐A‐treated NPC1L1‐OE C3H10 cells (H). (I) Representative western blot analysis of NPC1L1, С/ЕВРα, and Cyp27a1 in NPC1L1‐sh or NPC1L1‐OE osteoblasts. (J) Representative western blot analysis of NPC1L1, С/ЕВР, and Cyp27a1 in control, NPC1L1‐sh, and MTL‐C/EBPα‐treated NPC1L1‐sh osteoblast cells. All the data in the bar graphs are presented as the means ± SDs. *N* = 3 independent experiments. *p < 0.05; **p < 0.01; ***p < 0.001.

To clarify how NPC1L1 regulates cholesterol metabolism and 27‐OHC production, we examined the expression of Cyp27a1, a key enzyme that converts cholesterol to 27‐OHC. Compared with that in control cells, Cyp27a1 expression significantly increased during normal osteogenic differentiation (Figures 4F and 6A) but was downregulated in NPC1L1‐sh cells (Figure 6B). RT–qPCR confirmed that Cyp27a1 mRNA levels were elevated during osteogenesis but reduced following NPC1L1 knockdown, suggesting that Cyp27a1 mediates the effect of NPC1L1 on 27‐OHC generation during osteogenic differentiation (Figure 6C,D). To further validate this mechanism, we generated NPC1L1‐overexpressing (NPC1L1‐OE) osteoblasts and performed osteogenic induction and staining experiments. As expected, NPC1L1 overexpression inhibited osteogenesis; however, treatment with Dafadine‐A, a specific inhibitor of Cyp27a1, markedly reversed this inhibitory effect (Figures 6E,F and S3). Similarly, the reduced mRNA expression levels of osteogenic biomarkers in NPC1L1‐OE cells were restored to nearly normal levels by Dafadine‐A treatment, further suggesting that NPC1L1 enhances 27‐OHC production through regulating Cyp27a1 expression (Figure 6G,H). Using the promoter region sequence of Cyp27a1, we predicted potential upstream transcription factors through the JASPAR and UCSC databases. Combining these predictions with our RNA‐seq results, we hypothesized that C/EBPα likely mediates the regulatory effect of NPC1L1 on Cyp27a1 expression. Chromatin immunoprecipitation (ChIP)‐qPCR assays using control and NPC1L1‐OE osteoblasts verified that C/EBPα binds directly to the promoter region of Cyp27a1, thus increasing its expression. Western blot analysis further confirmed that NPC1L1 increases C/EBPα protein levels, thereby increasing Cyp27a1 expression and ultimately increasing 27‐OHC levels, resulting in the inhibition of osteogenic differentiation (Figure 6I). Finally, a rescue experiment using MTL‐C/EBPα restored C/EBPα expression in NPC1L1‐sh osteoblasts, which correspondingly increased the previously reduced Cyp27a1 levels (Figure 6J). Taken together, these findings demonstrate that the transcription factor C/EBPα mediates NPC1L1‐induced Cyp27a1 expression, leading to increased 27‐OHC production and subsequent inhibition of osteogenesis.

## 4 Discussion

Cholesterol is a key component of cellular membranes and serves as a precursor for synthesizing steroids, oxysterols, and bile acids, thus participating in various physiological processes within cells and the human body. Recently, increasing evidence has suggested that cholesterol metabolism plays a regulatory role in bone formation and osteogenic differentiation, although the exact mechanisms involved remain unclear [25]. For example, loss of Sc5d (Sc5d−/−), an enzyme that converts lathosterol into 7‐dehydrocholesterol, leads to cleft palate, micrognathia, and abnormal limb formation. Sc5d−/− mice specifically exhibit impaired osteogenic differentiation and mandibular hypoplasia [26]. Similarly, reduced expression of Dhcr7, another enzyme involved in cholesterol biosynthesis, disrupts cholesterol metabolism, and impairs osteogenic differentiation [27]. These findings suggest an essential relationship between cholesterol metabolism and osteogenic differentiation. However, the precise role of cholesterol metabolism in osteogenic differentiation remains unclear.

In our previous research, we demonstrated the circadian regulation of osteogenesis in osteoporosis and performed RNA‐seq to identify potential molecular mechanisms [28]. As shown in Figure 1A, significant changes were observed in lipid and cholesterol metabolism, notably in the cholesterol transporter NPC1L1 (Figure 1B,C). These findings support a role for cholesterol metabolism in osteogenic differentiation and encourage further exploration of how NPC1L1 regulates cholesterol to influence osteogenesis.

NPC1L1 is well known for its crucial role in intestinal cholesterol absorption and biliary cholesterol resorption by mediating cellular cholesterol uptake. However, few studies have investigated the role of NPC1L1 in cholesterol metabolism in osteoblasts. We initially explored whether NPC1L1 regulates osteogenesis through cholesterol transport. Interestingly, Eze, an inhibitor that blocks NPC1L1‐mediated cholesterol transport, did not affect osteogenic differentiation. By comparing NPC1L1‐knockdown cells with control cells via RNA‐seq, we detected significant upregulation of cholesterol metabolism‐related pathways in control osteoblasts after osteogenic induction (Figure 4D), whereas these pathways were downregulated in NPC1L1‐knockdown cells (Figure 4E). These results confirm the association of cholesterol metabolism with osteogenic differentiation and suggest that NPC1L1 influences osteogenesis by regulating cholesterol metabolism independently of its transport function. Currently, studies on NPC1L1 have focused largely on intestinal and hepatic cholesterol metabolism, and no reports have described its osteogenic regulatory function. Thus, our study reveals the previously unknown role of NPC1L1 in osteogenic differentiation and osteoporosis progression.

Oxysterols, derivatives of cholesterol found throughout the body, have diverse biological functions. For example, 7α‐hydroxycholesterol (7α‐OHC), produced by Cyp7a1, initiates bile acid synthesis; 24(S)‐hydroxycholesterol (24‐OHC) maintains cholesterol homeostasis in the brain; and 20(S)‐hydroxycholesterol (20S‐OHC) activates Hedgehog signaling, promoting osteogenic differentiation, while inhibiting adipogenesis in BMSCs. Notably, 27‐hydroxycholesterol (27‐OHC), generated by the mitochondrial enzyme Cyp27a1, negatively regulates bone homeostasis by impairing bone formation and enhancing bone resorption through LXR activation and increasing the expression of the osteoblasts TNF‐α and RANKL [29, 30]. In addition, 27‐OHC reportedly elevates the level of cellular reactive oxygen species (ROS), inhibiting cell viability. ROS play dual roles in osteogenic differentiation: physiological ROS levels facilitate differentiation and proliferation, whereas excessive ROS accumulation causes damage, leading to apoptosis and autophagy. In our study, we measured oxidative stress during osteogenesis via DCFH‐DA and confirmed increased ROS. Further tests revealed that 27‐OHC was significantly increased during osteogenesis but was only minimally increased in NPC1L1‐knockdown osteoblasts (Figure 5A–C). Conversely, the addition of external 27‐OHC increased ROS levels in NPC1L1‐sh cells, inhibiting differentiation. These findings suggest that NPC1L1 promotes cholesterol metabolism, generating 27‐OHC, which increases ROS levels and inhibits osteogenic differentiation. Conversely, inhibiting NPC1L1 reduces 27‐OHC and promotes differentiation.

Cyp27a1, a cytochrome P450 enzyme, converts cholesterol to 27‐OHC and influences bone homeostasis. Compared with control Cyp27a1−/− mice, female Cyp27a1−/− mice present increased but thinner trabeculae [31, 32]. In addition, Cyp27a1 deficiency reportedly enhances osteogenic differentiation and bone loss [33]. However, the precise mechanisms by which Cyp27a1 modulates osteogenic differentiation warrant further investigation, which presumably involves controlling 27‐OHC production. Transcriptomic data from our osteogenic differentiation experiments revealed increased Cyp27a1 expression, which aligns with the 27‐OHC production trends (Figure 4F). Although no significant changes in Cyp27a1 were observed before or after induction in NPC1L1‐sh cells, overall Cyp27a1 expression remained significantly lower than that in the controls after induction. Moreover, Dafadine‐A, a specific Cyp27a1 inhibitor, reversed the NPC1L1‐induced inhibition of osteogenesis, strongly supporting the hypothesis that NPC1L1 regulates osteogenesis via Cyp27a1‐driven 27‐OHC production. C/EBPα, a major regulator of adipogenesis, controls the osteogenic‐adipogenic balance during osteoporosis. We hypothesized that NPC1L1 enhances Cyp27a1 transcription via C/EBPα. Our data demonstrate that C/EBPα directly binds to the Cyp27a1 gene and enhances its expression, whereas NPC1L1 significantly promotes C/EBPα expression. Although our findings suggest that NPC1L1 positively regulates C/EBPα levels, the precise mechanism underlying this regulation (e.g., transcriptional activation vs. posttranslational stabilization) remains to be elucidated. Importantly, rescue experiments revealed that ectopic expression of MTL‐C/EBPα in NPC1L1‐silenced cells restored Cyp27a1 expression to baseline levels, further supporting the functional hierarchy of NPC1L1→C/EBPα→Cyp27a1. Additionally, cholesterol transport‐related genes (such as ABCA1 and ABCG1) and cholesterol metabolism pathway genes (such as HMGCR and SREBP‐2) may be involved in bone metabolism. However, these genes did not significantly change according to our RNA‐seq analysis, and preliminary experiments provided no clear evidence supporting their strong association with osteogenic differentiation. In future studies, we plan to systematically compare the roles of NPC1L1 and other cholesterol‐related molecules (e.g., ABCA1, SCARB1, HMGCR, and SREBP‐2) in bone differentiation. Moreover, owing to technical limitations, this study did not measure total cholesterol levels directly or HMGCR protein expression. Future work will focus on quantifying cholesterol metabolic flux and comparing the relative functional contributions of NPC1L1 and ABCA1/ABCG1, aiming to comprehensively understand the relationship between the cholesterol metabolic network and osteogenic differentiation.

## 5 Conclusion

This study reveals a previously unknown role of NPC1L1 in bone homeostasis as an important inhibitor of osteogenesis. We demonstrated that NPC1L1 suppresses bone formation by promoting the synthesis of 27‐OHC through the transcriptional activation of Cyp27a1 by C/EBPα. Our findings establish the NPC1L1/C/EBPα/Cyp27a1/27‐OHC axis as a key mechanism underlying cholesterol‐mediated inhibition of osteogenesis and osteoporosis development. This insight not only advances our understanding of metabolic regulation in skeletal biology but also identifies NPC1L1 and its downstream targets as promising therapeutic candidates. Pharmacological interventions targeting this axis, such as the use of NPC1L1 inhibitors or Cyp27a1 antagonists, may provide novel strategies to prevent or treat bone loss associated with osteoporosis. Future studies should assess the translational efficacy of these targets in preclinical models and explore interactions with other cholesterol‐related pathways (e.g., ABCA1, HMGCR) to comprehensively define the metabolic networks influencing bone health.

## Author Contributions

**Bohao Li:** Conceptualized the study, designed the methodology, conducted the investigation, performed formal analysis and data visualization, and wrote the original draft. **Wuling Zhou:** Conducted RNA‐seq data analysis (Figures 4F, and 5C), performed validation experiments, reviewed, and revised the manuscript text and figures. **Yueming Yu:** Supervised experimental design, contributed to data interpretation, and reviewed/edited the manuscript. **Boyu Chen:** Designed methodology, conducted investigations, curated data, and reviewed/edited the manuscript. **Zhicheng Lv:** Participated in experimental investigations and data curation. **Jiarui Zhang:** Provided critical reagents and resources, contributed to experimental investigations. **Tieqi Zhang:** Performed validation experiments and provided resources. **Shiwei Sun:** Managed project resources and coordination. **Lei Zhou:** Supervised the study, reviewed/edited the manuscript, and acquired funding. **Minghai Wang:** Supervised the overall project, managed administration, and acquired funding.

## Conflicts of Interest

The authors declare no conflicts of interest.

## Supporting information


Figures S1–S4.

Data S1.

## Data Availability

The RNA‐seq data analyzed in this study (GSE230665) are publicly available in the NCBI Gene Expression Omnibus (GEO) repository at https://www.ncbi.nlm.nih.gov/geo/query/acc.cgi?acc=GSE230665. All other raw data supporting the findings of this study, including experimental datasets and analytical outputs, are available from the corresponding authors upon reasonable request.

## References

[1] J. He , S. Xu , B. Zhang , et al., “Gut Microbiota and Metabolite Alterations Associated With Reduced Bone Mineral Density or Bone Metabolic Indexes in Postmenopausal Osteoporosis,” Aging (Albany NY) 12, no. 9 (2020): 8583–8604. 32392181 10.18632/aging.103168 PMC7244073

[2] M. Das , O. Cronin , D. M. Keohane , et al., “Gut Microbiota Alterations Associated With Reduced Bone Mineral Density in Older Adults,” Rheumatology (Oxford) 58, no. 12 (2019): 2295–2304. 31378815 10.1093/rheumatology/kez302 PMC6880854

[3] D. M. Black and C. J. Rosen , “Clinical Practice. Postmenopausal Osteoporosis,” New England Journal of Medicine 374, no. 3 (2016): 254–262. 26789873 10.1056/NEJMcp1513724

[4] L. G. Raisz , “Pathogenesis of Osteoporosis: Concepts, Conflicts, and Prospects,” Journal of Clinical Investigation 115, no. 12 (2005): 3318–3325. 16322775 10.1172/JCI27071 PMC1297264

[5] T. L. Yang , H. Shen , A. Liu , et al., “A Road Map for Understanding Molecular and Genetic Determinants of Osteoporosis,” Nature Reviews. Endocrinology 16, no. 2 (2020): 91–103. 10.1038/s41574-019-0282-7 PMC6980376 31792439

[6] R. Eastell and P. Szulc , “Use of Bone Turnover Markers in Postmenopausal Osteoporosis,” Lancet Diabetes and Endocrinology 5, no. 11 (2017): 908–923. 28689768 10.1016/S2213-8587(17)30184-5

[7] F. Long , “Energy Metabolism and Bone,” Bone 115 (2018): 1, 10.1016/j.bone.2018.08.002. 30146067

[8] J. O. Nehlin , A. Jafari , M. Tencerova , and M. Kassem , “Aging and Lineage Allocation Changes of Bone Marrow Skeletal (Stromal) Stem Cells,” Bone 123 (2019): 265–273. 30946971 10.1016/j.bone.2019.03.041

[9] W. Yin , Z. Li , and W. Zhang , “Modulation of Bone and Marrow Niche by Cholesterol,” Nutrients 11, no. 6 (2019): 1394, 10.3390/nu11061394. 31234305 PMC6628005

[10] Y. Shin , Y. Won , J. I. Yang , and J. S. Chun , “CYTL1 Regulates Bone Homeostasis in Mice by Modulating Osteogenesis of Mesenchymal Stem Cells and Osteoclastogenesis of Bone Marrow‐Derived Macrophages,” Cell Death & Disease 10, no. 2 (2019): 47. 30718470 10.1038/s41419-018-1284-4 PMC6362050

[11] R. Cai , T. Nakamoto , T. Hoshiba , N. Kawazoe , and G. Chen , “Matrices Secreted During Simultaneous Osteogenesis and Adipogenesis of Mesenchymal Stem Cells Affect Stem Cells Differentiation,” Acta Biomaterialia 35 (2016): 185–193. 26873367 10.1016/j.actbio.2016.02.009

[12] A. Rauch , A. K. Haakonsson , J. G. S. Madsen , et al., “Osteogenesis Depends on Commissioning of a Network of Stem Cell Transcription Factors That Act as Repressors of Adipogenesis,” Nature Genetics 51, no. 4 (2019): 716–727. 30833796 10.1038/s41588-019-0359-1

[13] R. Yue , B. O. Zhou , I. S. Shimada , Z. Zhao , and S. J. Morrison , “Leptin Receptor Promotes Adipogenesis and Reduces Osteogenesis by Regulating Mesenchymal Stromal Cells in Adult Bone Marrow,” Cell Stem Cell 18, no. 6 (2016): 782–796. 27053299 10.1016/j.stem.2016.02.015

[14] D. Song , D. Liu , W. Ning , et al., “Incidence, Prevalence and Characteristics of Multimorbidity in Different Age Groups Among Urban Hospitalized Patients in China,” Scientific Reports 13, no. 1 (2023): 18798, 10.1038/s41598-023-46227-4. 37914899 PMC10620234

[15] X. H. Li , W. W. Pang , Y. Zhang , et al., “A Mendelian Randomization Study for Drug Repurposing Reveals Bezafibrate and Fenofibric Acid as Potential Osteoporosis Treatments,” Frontiers in Pharmacology 14 (2023): 1211302, 10.3389/fphar.2023.1211302. 37547327 PMC10397407

[16] M. Leutner , C. Matzhold , L. Bellach , et al., “Diagnosis of Osteoporosis in Statin‐Treated Patients Is Dose Dependent,” Annals of the Rheumatic Diseases 78, no. 12 (2019): 1706–1711. 31558481 10.1136/annrheumdis-2019-215714 PMC6900255

[17] L. You , Z. Y. Sheng , C. L. Tang , et al., “High Cholesterol Diet Increases Osteoporosis Risk by Inhibiting Bone Formation in Rats,” Acta Pharmacologica Sinica 32, no. 12 (2011): 1498–1504. 22036861 10.1038/aps.2011.135 PMC4010216

[18] Y. S. Seo , K. R. Kang , H. Lim , et al., “25‐Hydroxycholesterol‐Induced Osteoblast Oxiapoptophagy Is Involved in the Pathophysiological Process of Osteoporosis,” In Vivo 37, no. 1 (2023): 204–217. 36593033 10.21873/invivo.13069 PMC9843796

[19] R. Cui , L. Zhou , Z. Li , Q. Li , Z. Qi , and J. Zhang , “Assessment Risk of Osteoporosis in Chinese People: Relationship Among Body Mass Index, Serum Lipid Profiles, Blood Glucose, and Bone Mineral Density,” Clinical Interventions in Aging 11 (2016): 887–895. 27445467 10.2147/CIA.S103845 PMC4938238

[20] B. A. Ference , F. Majeed , R. Penumetcha , J. M. Flack , and R. D. Brook , “Effect of Naturally Random Allocation to Lower Low‐Density Lipoprotein Cholesterol on the Risk of Coronary Heart Disease Mediated by Polymorphisms in NPC1L1, HMGCR, or Both: A 2 × 2 Factorial Mendelian Randomization Study,” Journal of the American College of Cardiology 65, no. 15 (2015): 1552–1561. 25770315 10.1016/j.jacc.2015.02.020 PMC6101243

[21] N. O. Stitziel , H. H. Won , A. C. Morrison , et al., “Inactivating Mutations in NPC1L1 and Protection From Coronary Heart Disease,” New England Journal of Medicine 371, no. 22 (2014): 2072–2082, 10.1056/NEJMoa1405386. 25390462 PMC4335708

[22] R. Li , Y. Liu , J. Shi , et al., “Diosgenin Regulates Cholesterol Metabolism in Hypercholesterolemic Rats by Inhibiting NPC1L1 and Enhancing ABCG5 and ABCG8,” Biochimica et Biophysica Acta ‐ Molecular and Cell Biology of Lipids 1864, no. 8 (2019): 1124–1133. 31054325 10.1016/j.bbalip.2019.04.010

[23] C. Xu , F. Fu , Y. She , and C. Xu , “NPC1L1 Plays a Novel Role in Nonalcoholic Fatty Liver Disease,” ACS Omega 8, no. 51 (2023): 48586–48589. 38162748 10.1021/acsomega.3c07337 PMC10753569

[24] J. Lin , W. Q. Shao , Q. Z. Chen , et al., “Osteopontin Deficiency Protects Mice From Cholesterol Gallstone Formation by Reducing Expression of Intestinal NPC1L1,” Molecular Medicine Reports 16, no. 2 (2017): 1785–1792. 28627641 10.3892/mmr.2017.6774 PMC5561929

[25] C. Iwaya , A. Suzuki , J. Shim , A. Kim , and J. Iwata , “Craniofacial Bone Anomalies Related to Cholesterol Synthesis Defects,” Scientific Reports 14, no. 1 (2024): 5371. 38438535 10.1038/s41598-024-55998-3 PMC10912708

[26] C. Iwaya , A. Suzuki , and J. Iwata , “Loss of Sc5d Results in Micrognathia due to a Failure in Osteoblast Differentiation,” Journal of Advanced Research 65 (2024): 153–165, 10.1016/j.jare.2023.12.008. 38086515 PMC11519736

[27] A. Suzuki , K. Ogata , H. Yoshioka , et al., “Disruption of Dhcr7 and Insig1/2 in Cholesterol Metabolism Causes Defects in Bone Formation and Homeostasis Through Primary Cilium Formation,” Bone Research 8 (2020): 1. 31934493 10.1038/s41413-019-0078-3 PMC6946666

[28] L. Zhou , T. Zhang , S. Sun , Y. Yu , and M. Wang , “Cryptochrome 1 Promotes Osteogenic Differentiation of Human Osteoblastic Cells via Wnt/β‐Catenin Signaling,” Life Sciences 212 (2018): 129–137. 30290183 10.1016/j.lfs.2018.09.053

[29] L. Zhang , M. Liu , J. Liu , et al., “27‐Hydroxycholesterol Enhanced Osteoclastogenesis in Lung Adenocarcinoma Microenvironment,” Journal of Cellular Physiology 234, no. 8 (2019): 12692–12700. 30511368 10.1002/jcp.27883 PMC13484145

[30] E. R. Nelson , C. D. Dusell , X. Wang , et al., “The Oxysterol, 27‐Hydroxycholesterol, Links Cholesterol Metabolism to Bone Homeostasis Through Its Actions on the Estrogen and Liver X Receptors,” Endocrinology 152, no. 12 (2011): 4691–4705. 21933863 10.1210/en.2011-1298 PMC3230052

[31] P. Orozco , “Atherogenic Lipid Profile and Elevated Lipoprotein (a) Are Associated With Lower Bone Mineral Density in Early Postmenopausal Overweight Women,” European Journal of Epidemiology 19, no. 12 (2004): 1105. 15678790 10.1007/s10654-004-1706-8

[32] L. B. Tankó , Y. Z. Bagger , S. B. Nielsen , and C. Christiansen , “Does Serum Cholesterol Contribute to Vertebral Bone Loss in Postmenopausal Women?,” Bone 32, no. 1 (2003): 8–14. 12584030 10.1016/s8756-3282(02)00918-3

[33] Z. Fang , G. Cheng , M. He , and Y. Lin , “CYP27A1 Deficiency Promoted Osteoclast Differentiation,” PeerJ 11 (2023): e15041. 36890868 10.7717/peerj.15041 PMC9987298

