# Supplementary material for: NPC1L1 Drives Osteoporosis by Activating the C/EBPα/Cyp27a1/27‐Hydroxycholesterol Axis: A Novel Therapeutic Target for Bone Loss
Source: FASEB Bioadv. 2025 May 8;7(6):e70020. doi: 10.1096/fba.2025-00044 (PMC12147501; doi:10.1096/fba.2025-00044)
Supplement: Supplementary file 1 — Figures S1–S4. [file FBA2-7-e70020-s001.docx]

Mechanism of NPC1L1 mediated 27-hydroxycholesterol metabolisms in the occurrence and development of osteoporosis

**Supplementary Figures**


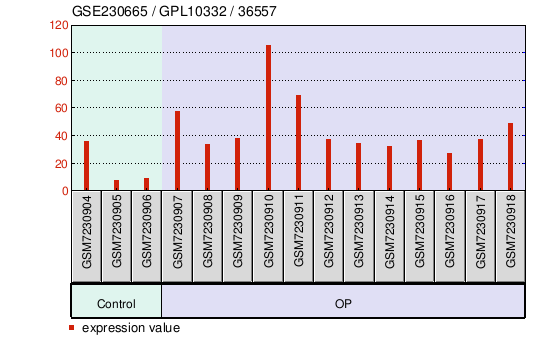


Figure S1: Relative NPC1L1 expression levels in femur of postmenopausal osteoporosis patients (OP), compared with 3 healthy postmenopausal women as control group.


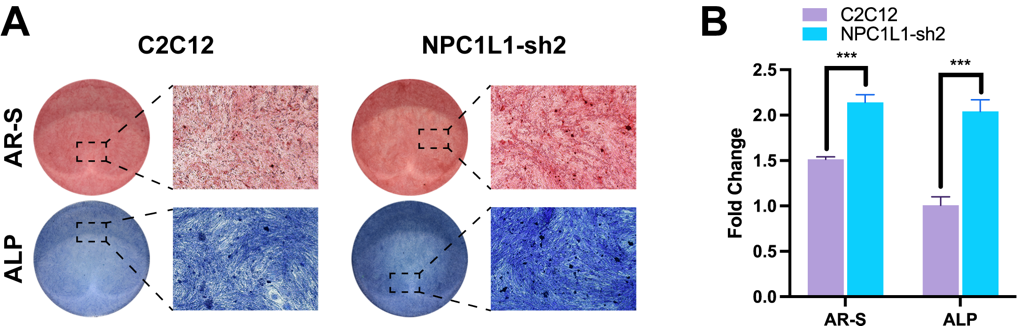


Figure S2: A. AR-S (upper panel) and ALP (lower panel) staining of blank and NPC1L1-sh2 C2C12 cells after induction with osteogenic medium for 3 days. B. Quantitative analysis of AR-S and ALP staining results. All bar graphs are presented as the mean ± SD. *P < 0.05; **P < 0.01; ***P < 0.001.


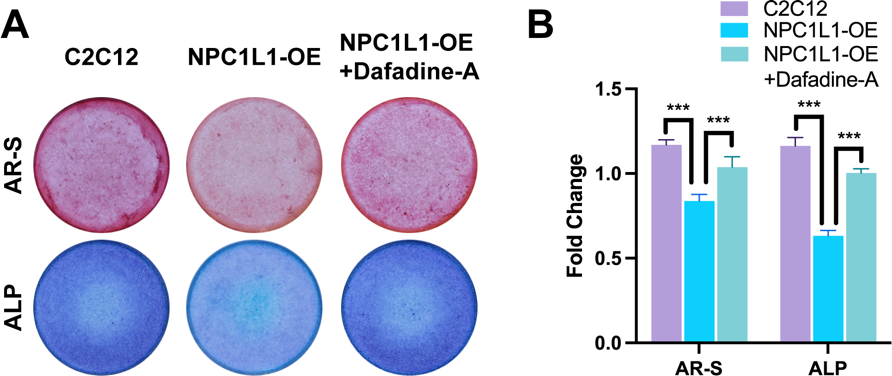


Figure S3: A. AR-S (upper panel) and ALP (lower panel) staining of control, NPC1L1 overexpression (NPC1L1-OE), and Dafadine-A treated NPC1L1-OE C2C12 cells after induction with osteogenic medium for 3 days. B. Quantitative analysis of AR-S and ALP staining results. All bar graphs are presented as the mean ± SD. *P < 0.05; **P < 0.01; ***P < 0.001.


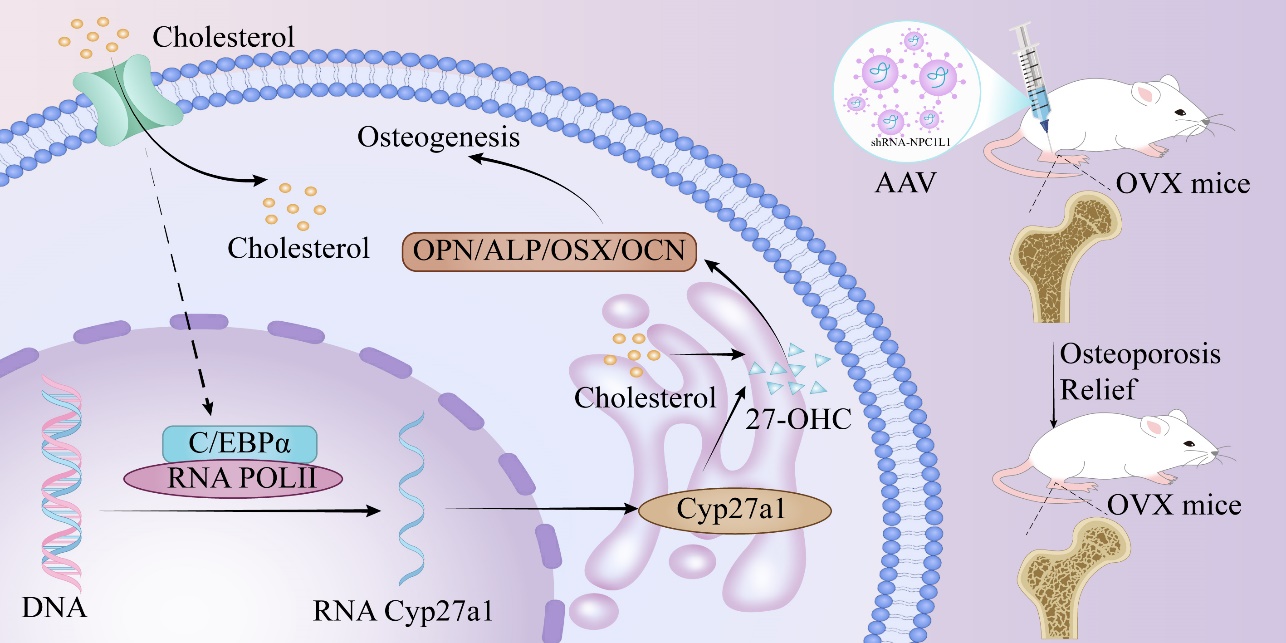


Figure S4: Graphical Abstract
