# Supplementary figures and images for: NPC1L1 Drives Osteoporosis by Activating the C/EBPα/Cyp27a1/27‐Hydroxycholesterol Axis: A Novel Therapeutic Target for Bone Loss
Source: FASEB Bioadv. 2025 May 8;7(6):e70020. doi: 10.1096/fba.2025-00044 (PMC12147501; doi:10.1096/fba.2025-00044)

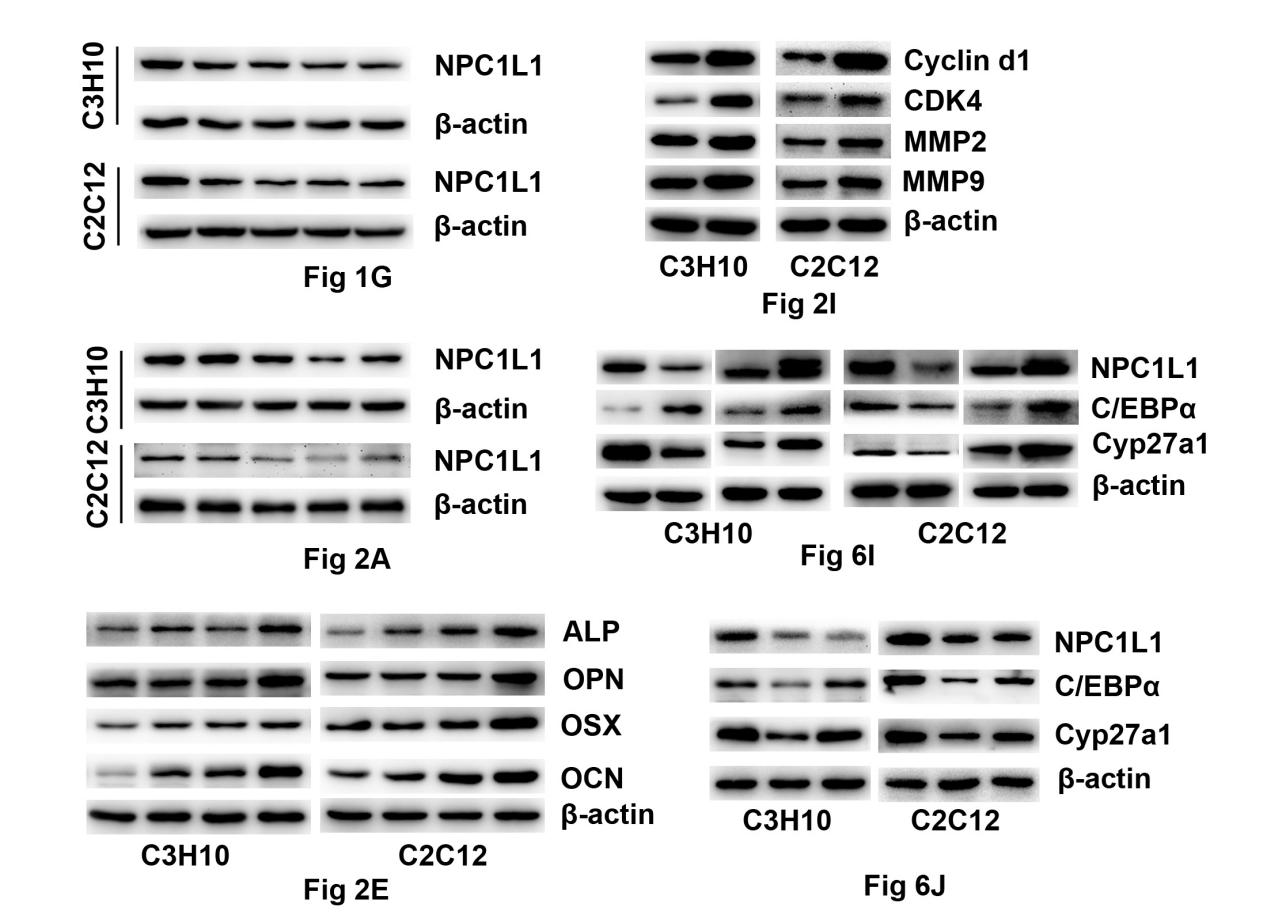

Supplement: Supplementary file 2 — Data S1. [file FBA2-7-e70020-s002.docx]
